# Transcriptome Analysis of Indole-3-Butyric Acid-Induced Adventitious Root Formation in Nodal Cuttings of *Camellia sinensis* (L.)

**DOI:** 10.1371/journal.pone.0107201

**Published:** 2014-09-12

**Authors:** Kang Wei, Li-Yuan Wang, Li-Yun Wu, Cheng-Cai Zhang, Hai-Lin Li, Li-Qiang Tan, Hong-Li Cao, Hao Cheng

**Affiliations:** 1 Key Laboratory of Tea Biology and Resources Utilization, Ministry of Agriculture, National Center for Tea Improvement, Hangzhou, PR China; 2 National Center for Tea Improvement, Tea Research Institute Chinese Academy of Agricultural Sciences (TRICAAS), Hangzhou, PR China; Henan Agricultural Univerisity, China

## Abstract

Tea (*Camellia sinensis* L.) is a popular world beverage, and propagation of tea plants chiefly depends on the formation of adventitious roots in cuttings. To better understand potential mechanisms involved in adventitious root formation, we performed transcriptome analysis of single nodal cuttings of *C. sinensis* treated with or without indole-3-butyric acid (IBA) using the Illumina sequencing method. Totally 42.5 million RNA-Seq reads were obtained and these were assembled into 59,931 unigenes, with an average length of 732 bp and an N50 of 1292 bp. In addition, 1091 differentially expressed unigenes were identified in the tea cuttings treated with IBA compared to controls, including 656 up- and 435 down-regulated genes. Further real time RT-PCR analysis confirmed RNA-Seq data. Functional annotation analysis showed that many genes were involved in plant hormone signal transduction, secondary metabolism, cell wall organization and glutathione metabolism, indicating potential contributions to adventitious rooting. Our study presents a global view of transcriptome profiles of tea cuttings in response to IBA treatment and provides new insights into the fundamental mechanisms associated with auxin-induced adventitious rooting. Our data will be a valuable resource for genomic research about adventitious root formation in tea cuttings, which can be used to improve rooting for difficult-to-root varieties.

## Introduction

Tea (*Camellia sinensis* L.) is one of the most popular beverages in the world. The popularity of tea is not only due to its rich flavor and taste, but also associated with many beneficial effects of human being, such as cancer protection [1] and anti-obesity effects [2]. At this time, tea plant propagation is mainly through nodal cuttings, which ensure quality stability and facilitate the spread of improved cultivars. However, this method relies on the formation of adventitious roots (ARs) and plant losses due to slow or non-rooted cuttings frequently happen in agricultural practice. Therefore, AR formation is considered a prerequisite for successful propagation of tea cuttings.

AR formation is a complex regeneration process with many internal and external factors influencing AR formation, including environmental conditions, phytohormones and nutrition status [3]–[5]. Among them, the phytohormone auxin is crucial, as it promotes AR formation in cuttings [6]. Meanwhile, many factors affect AR formation via auxin interactions. For example, ethylene, another stimulator of AR formation, may regulate auxin transport [7]. While, light control of AR formation is via auxin homeostasis perturbation [8]. Therefore, the auxin-associated mechanism appears essential for AR formation. However, mechanisms behind this process are only superficially understood, thereby limiting improvements to cutting propagation.

Indole-3-butyric acid (IBA) is widely used in tea propagation to induce rooting. Although indole-3-acetic acid (IAA) is a primarily native auxin in plants, IBA is more effective in promoting adventitious roots [9]–[11]. Tea cuttings pre-treated with IBA showed significantly better rooting ability than controls after being transferred to a potting medium [10]. Furthermore, root initiation of tea cuttings pre-treated with IBA occurred much earlier than controls, suggesting that IBA-regulated genes might directly affect the root induction process and other studies support this assertion. For example, Brinker *et al.* (2004) reported that IBA induced expression of genes involved in cell replication and cell wall weakening, while inhibited genes related to auxin transport, photosynthesis and cell wall synthesis during the root initiation of *Pinus contorta* [12]. Ludwig-Müller’s group (2005) found that a protein phosphatase 2A gene might be important for IBA-induced AR formation on *Arabidopsis* stem segments [9]. Thus, understanding processes that occur in cuttings after IBA treatment is necessary, especially functions of IBA-regulated genes. However, at this time, IBA-regulated genes in tea cuttings are still poorly understood. We previously identified 77 differentially expressed transcripts in tea cuttings treated with or without IBA by suppressive subtractive hybridization (SSH) [11] and this represented the first exploration of potential genes involved in AR formation in the tea plant. However, SSH method is insufficient for global characterization of differentially expressed genes. Furthermore, it does not provide quantitative expression of candidate genes and may contain false positives [13]. The RNA-Seq method of high-throughput sequencing technology for discovering differentially expressed genes largely overcomes these limitations [14]–[16]. Here, we used RNA-Seq for tea cuttings treated with or without IBA (NCBI BioProject Accession: PRJNA240661, http://www.ncbi.nlm.nih.gov/bioproject/240661) to identify new genes involved in IBA-induced AR formation and gain a deeper insight into the mechanism of tea propagation. Moreover, the application of RNA-Seq will also provide more transcripts to facilitate further genomic studies of *C. sinensis*.

## Materials and Methods

### Plant Material

Freshly growing twigs of *C. sinensis* var. Longjing 43 were collected from the tea garden of Tea Research Institute Chinese Academy of Agricultural Sciences, Hangzhou, China and transported to the laboratory in a water cool box. Each twig was further segmented into smaller pieces resulting in one node with a single leaf in each segment. The single node cuttings of uniform size were selected and placed in 1000 ml glass beakers containing 200 ml basic nutrient solution (1/10 Hoagland nutrient solution). The basal region of the nodal cuttings was dipped into the solution (pH 5.8) [12]. IBA was then added to corresponding beakers to form 2 treatments: (1) control only containing the basic solution; (2) IBA treatment (0.4 mM) for 24 h, which was modified from the experimental design of Wei (2013) [11]. Stem samples of the IBA treatment and controls were then harvested. Basal parts (about 1.0 cm of the root zone) of the cuttings were taken, frozen immediately in liquid nitrogen, and stored at –80°C for subsequent analysis.

### RNA Extraction, cDNA Library Construction, and Sequencing

Total RNAs were extracted from stem samples with or without IBA treatment using TriReagent (Qiagen, Valencia, CA), and the mRNAs were purified from the total RNAs using the Truseq RNA Sample Prep Kit (Illumina) following the manufacturer’s instructions. Briefly, mRNA was isolated from >5 µg of total RNA using oligo (dT) magnetic beads. Integrity and size distribution were confirmed with Bioanalyzer 2100 (Agilent technologies, Palo Alto, CA, USA). mRNA was cut into short fragments by adding fragmentation buffer. First-strand cDNA was synthesized using random hexamer-primers, taking these short fragments as templates. RNaseH, buffer, dNTPs, and DNA polymerase I was used to synthesize second-strand cDNA. Short fragments were purified with Takara’s PCR extraction kit (Takara Bio, Otsu, Japan). Sequencing adapters were ligated to short fragments and resolved by agarose gel electrophoresis. Proper fragments were selected and purified and subsequently PCR amplified to create the final cDNA library template.

The cDNA was then shotgun sequenced (101-bp paired-end read) with the Illumina HiSeq 2000 instrument (Illumina, San Diego, CA, USA) using a customer sequencing service (Biomarker Technologies Co., Ltd, Beijing, China) which also included nebulization and end repair of cDNA, ligation of adaptors, gel purification, PCR amplification and library purification.

### *De novo* Assembly and Functional Annotation

The raw reads were cleaned by removing adaptor sequences, empty reads, and low-quality sequences (reads with unknown sequences ‘N’ or less than 25 bp). Clean reads were assembled into non-redundant transcripts using Trinity, which has been developed specifically for *de novo* assembly of transcriptomes using short reads. Short sequences (<200 bp in length) were also removed to improve quality. Resulting sequences were used for BLAST searches and annotation against NCBI non-redundant protein (NR), nucleotide collection (NT), Swissprot, TrEMBL, Clusters of Orthologous Groups of proteins (COG), and Kyoto Encyclopedia of Genes and Genomes (KEGG) databases using an E-value cut-off of 10^−5^. Functional annotation by gene ontology (GO) terms (www.geneontology.org) was analyzed by the Blast2go software.

### Identification of Differentially Expressed Genes

For differential gene expression analysis, RPKM (reads per kilobase per million reads) was used as a value of normalized gene expression [17]. Statistical comparison of RPKM values between the IBA treated and control samples was conducted using a web tool IDEG6 (http://telethon.bio.unipd.it/bioinfo/IDEG6_form/) [18]. A false discovery rate (FDR) of 0.01 was used as the threshold of P-value in multiple test to evaluate the significance of gene expression differences. Genes were considered differentially expressed in a given library when P-value <0.01 and a greater than two-fold change in expression across libraries was observed. Furthermore, GO classifications were compared for up- and down-regulated unigenes using WEGO [19]. COG and KEGG pathway annotations were also performed using Blastall software against COG and KEGG databases.

### Real Time RT-PCR Analysis

To validate Illumina sequencing data, 36 differentially expressed genes were selected for real time RT-PCR analysis, using new RNA isolated from four biological replicate stem samples. RNA isolation was done according to a modified CTAB method described by Chen *et al.* (2012) [20]. Primers were designed using Primer 3 software to amplify 150–250 bp fragments for 36 transcripts selected from the RNA-Seq libraries as potential candidate genes (Table S1). Real time expression assays were performed with SYBR Green dye (Sigma-Aldrich) on the ABI 7500 Real-Time PCR System (Applied Biosystems). 18S ribosomal RNA (AB120309.1, *C. sinensis*) was utilized as the house-keeping gene. PCR reaction efficiency was calculated for each primer set using serial dilutions of a cDNA template and plotting Ct values against the log of the template concentrations. The slope of the line was subsequently used to calculate the amplification efficiency (E) according to: E = 10(–1/slope), which was used in the calculation of relative normalized expression in QBase [21].

## Results

### RNA-Seq

To obtain genes involved in IBA-induced adventitious root formation, we used basal parts of tea cuttings treated with or without IBA treatment prior to RNA-Seq. The two cDNA libraries obtained from the control and IBA treated samples were subjected to sequencing by the Illumina HiSeq2000 genome analyzer. Totally 42.537 million reads and a Q20 percentage (percentage of sequences with sequencing error rate lower than 1%) over 89% were generated (Table 1). These data were then deposited in the National Center for Biotechnology Information (NCBI) with accession number of PRJNA240661 (http://www.ncbi.nlm.nih.gov/bioproject/240661). After trimming low-quality sequences, *de novo* assembly was performed with combined cleaned reads from both libraries by the software Trinity. Finally, short reads were assembled into 59,931 unigenes, with an average length of 732 bp and N50 length of 1292 bp (Table 2).

**Table 1:** Summary for RNA-Seq datasets of *C. sinensis.*

	Number of reads (million)	Total base (Gb)	Q20 percentage
Control	21.265	4.295	89.15%
IBA treatment	21.272	4.297	89.02%

**Table 2:** Length distribution of assembled unigenes.

Unigenes Length (bp)	Number of sequences	Percentage
200–300	20705	34.55%
300–500	16313	27.22%
500–1000	10541	17.59%
1000–2000	7600	12.68%
2000+	4772	7.96%
Total number	59931	
Average length	732 bp	
N50 length	1292 bp	

### Functional Annotation

Most (51%) of the unigenes (30,558) gave BLASTx hits against 7 public databases (the COG, GO, KEGG, Swiss-Prot, TrEMBL, NR and NT databases) with an E-value threshold of 10^−5^ (Table 3). Among them, most unigenes were annotated to the TrEMBL (28,372) and NR (28,318) databases. Wang *et al.* (2013) reported that 97.5% of the annotated unigenes were annotated to the NR database [22], indicating that the NR database was an informatic platform for functional annotation of *C. sinensis*. Furthermore, 41% of the NR annotated unigenes had strong homology to *Vitis vinifera* according to the NR annotation, which was greater than other species (Figure S1). The high sequence similarity between *C. sinensis* and *Vitis vinifera* may explain their high flavonoid accumulation, which is worthy of further study [23], [24].

**Table 3:** Summary for the BLASTx results of *C. sinensis* transcriptome against seven databases.

Annotation database	Annotated Number
COG Annotation	7729
GO Annotation	22360
KEGG Annotation	5131
Swissprot Annotation	18046
TrEMBL Annotation	28372
NR Annotation	28318
NT Annotation	23857
All Annotated unigenes	30558
Unigenes hit all seven databases	2175

GO analysis was also performed (see Figure S2) and GO-annotated unigenes could be divided into cellular component, molecular function and biological process clusters. Among the cellular components category, cell part (28.9%), cell (28.5%) and organelle (25.6%) were the dominant groups. In terms of molecular functions, binding (22.1%) and catalytic activity (20.1%) were the dominant groups. Most biological process genes were involved in cellular processes (27.6%) and metabolic processes (27.5%).

We also searched the unigenes against COG and KEGG databases for functional prediction and classification. Among the 25 COG categories, most unigenes were classified into the cluster for “general function prediction only”, followed by the “replication, recombination and repair” and “transcription” (Figure S3). The high number of unigenes with only predicted functions suggests many genes in *C. sinensis* are novel and might have new functions which may not be identified in model plants. For KEGG analysis, 5131 unigenes were assigned to 117 KEGG pathways and the distribution of KEGG pathway is shown in Figure S4.

### Identification of Differentially Expressed Genes

To further explore differentially expressed genes involved in IBA-induced AR formation in tea cuttings, expression of unigenes in each library with RPKM were compared. Quantitative profiling of transcriptomes reveals that 1091 unigenes were differentially expressed, with 656 unigenes up-regulated and 435 down-regulated by IBA (Table S2). See Table S2 for differentially expressed unigenes and COG, GO, KEGG, Swiss-Prot, TrEMBL, NR and NT annotations.

To obtain a global view of IBA associated genes involved in AR formation, we mapped 1091 unigenes to reference canonical pathways based on KEGG database and 118 unigenes were assigned to 69 pathways (Table S3). Among them, 12 pathways were significantly affected by IBA (P-value<5%), indicating potential importance in IBA responses (Table S3). The most representative pathway was plant hormone signal transduction (Ko04075, 24 unigenes, P-value = 6.06 e^−10^), followed by zeatin biosynthesis (Ko00908, 6 unigenes, P-value = 5.77 e^−6^), cysteine and methionine metabolism (Ko00270, 11 unigenes, P-value = 5.03 e^−5^) and phenylalanine metabolism (Ko00360, 9 unigenes, P-value = 1.95 e^−4^). The classification indicates that the plant hormone signal transduction may be key to IBA-induced AR formation in *C. sinensis*.

**Figure 1:**
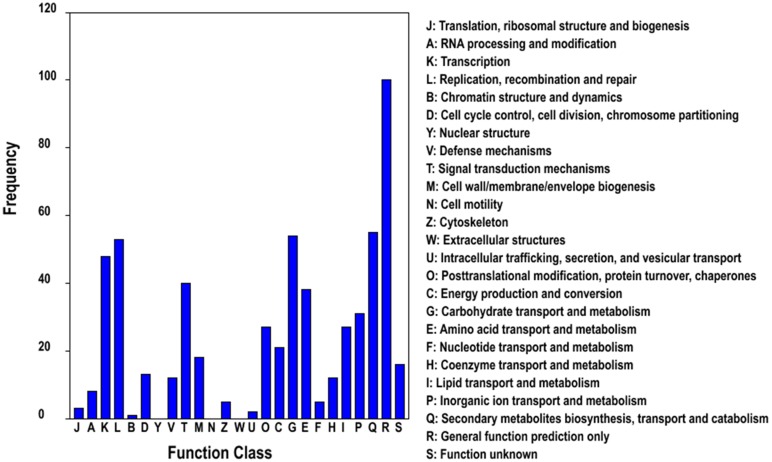
COG function classification of differentially expressed unigenes in *C. sinensis*.

Moreover, the COG classification of differentially expressed unigenes showed that most unigenes were classified as “general function prediction only”, which is similar to findings in the COG classification of all unigenes (Figures 1 and S3). The following categories include “secondary metabolites biosynthesis, transport and catabolism” (55 unigenes), “carbohydrate transport and metabolism” (54 unigenes), “replication, recombination and repair” (53 unigenes) and “transcription” (48 unigenes), indicating IBA exerts a broad range of effects on plant metabolism.

**Figure 2:**
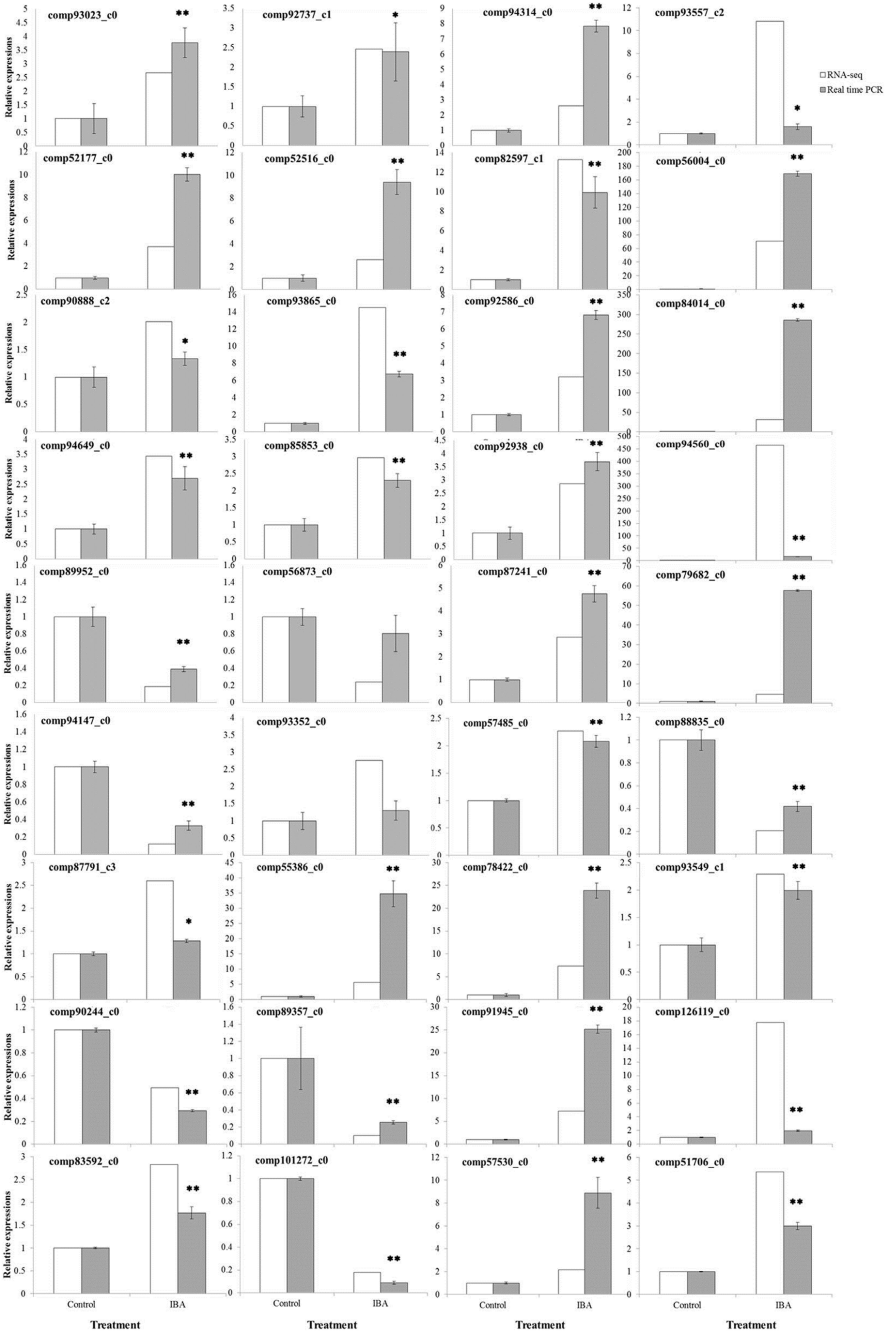
Comparison of the expression profiles of selected genes as determined by RNA-Seq and Real Time-PCR. Data of real time PCR analysis are the means ± standard errors (n = 4). * represents a significant difference between control and IBA treatment (P<0.05). ** represents a highly significant difference between control and IBA treatment (P<0.01).

On the other hand, 36 differentially expressed unigenes were selected to test our RNA-seq results with real time RT-PCR (Figure 2). These unigenes are related to plant hormone signal transduction, nitrogen metabolism, phenylalanine metabolism as well as other enzymatic processes (Table S1). Among them, 29 unigenes were up-regulated and 7 were down-regulated by IBA according to the RNA-seq results. Real time RT-PCR analysis indicated that expression of 34 unigenes (94.4%) were highly consistent with RNA-seq results, with the same changing trends and significant differences between IBA-treated samples and controls. Only cytokinin dehydrogenase (comp56873_c0) and cyanidin-3-O-glucoside 2-O-glucuronosyltransferase (comp93352_c0) exhibited the same changing directions but they were not significantly different. In summary, real time RT-PCR analysis agreed well with RNA-seq analysis, indicating that the RNA-seq method could identify differentially expressed genes.

### Redundant Genes Involved in IBA Response

**Figure 3:**
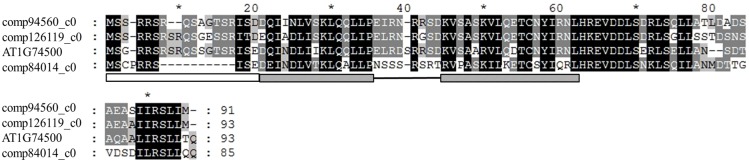
Multiple alignment of amino acid sequences of comp94560_c0, comp126119_c0, comp84014_c0 (*C. sinensis*) and their homologous gene AT1G74500 (*Arabidopsis thaliana*) was performed with CLUSTALX 1.83. Identical and similar amino acids are shaded black and gray, respectively. Open box denotes the basic region, hatched bar indicates the helix motif, and the black line represents the loop region.

Differentially expressed unigenes were also analyzed with BLASTx against *Arabidopsis* proteome sequences. Interestingly, many redundant genes were found (redundant gene number ≥3; see Table 4). Redundant genes had the same trend of change with IBA treatment, indicating regulation via similar mechanisms. Of note, 4 unigenes were homologues to AT1G74500, namely comp94560_c0, comp56004_c0, comp126119_c0 and comp84014_c0, whose expressions were 465.1-, 70.6-, 17.8- and 31.3- fold respectively induced by IBA, a greater expression than other redundant genes. Multiple sequence alignment of amino acids of those genes is offered in Figure 3. The comp56004_c0 was not included in the alignment as it contained only part of the open reading frame. The comp94560_c0, comp126119_c0, comp84014_c0 and AT1G74500 genes shared about 71, 70 and 56% amino acid sequence identity, respectively. Thus, these genes likely have a similar function to AT1G74500. *Arabidopsis* gene (AT1G74500) encodes the basic helix-loop-helix protein 135 (*bHLH135*), which is essential to regulate brassinosteroid (BR) signaling [25]. Therefore, BR signaling might be also involved in auxin response in tea cuttings.

**Table 4:** The information of redundant unigenes in the differentially expressed unigenes of tea cuttings treated with or without IBA.

Homologous genein Arabidopsis	Functional description	Accession	Redundant gene number	Regulation by IBA treatment
AT1G78380	Glutathione S-transferase *TAU 19*	NP_565178	6	Induction
AT1G74500	Transcription factor *bHLH135*	NP_177590	4	Induction
AT5G01240	Auxin transporter-like protein 1	NP_974719	3	Inhibition
AT3G53210	Nodulin MtN21/EamA-like transporter family protein	NP_566981	3	Induction
AT5G13000	Callose synthase 3	NP_001154712	3	Induction
AT4G37390	Indole-3-acetic acid-amido synthetase GH3.2	NP_195455	3	Induction
AT5G65730	Xyloglucan endotransglucosylase/hydrolase protein	NP_569019	3	Inhibition

## Discussion

We report a comparative transcriptome analysis of tea cuttings treated with or without IBA using RNA-seq and we observed that various genes and pathways are potentially responsible for AR formation. In total, 1091 differentially expressed unigenes were identified, a larger set than our previous work with the SSH method [11]. Meanwhile, differentially expressed unigenes identified with RNA-seq and previous SSH were compared, and only 7 unigenes overlapped. Most unigenes identified with SSH were in the range of 1.2 to 2 times the expression difference according to RNA-seq results, indicating that the RNA-seq method is more powerful for identifying highly differentially expressed unigenes. Furthermore, the presence of more differentially expressed genes sheds light on a global view of IBA-induced AR formation in tea cuttings, which may assist us to understand molecular mechanisms behind this process as well as facilitate rooting efficiency in agricultural practice.

### Plant Hormone Signal Transduction

**Figure 4:**
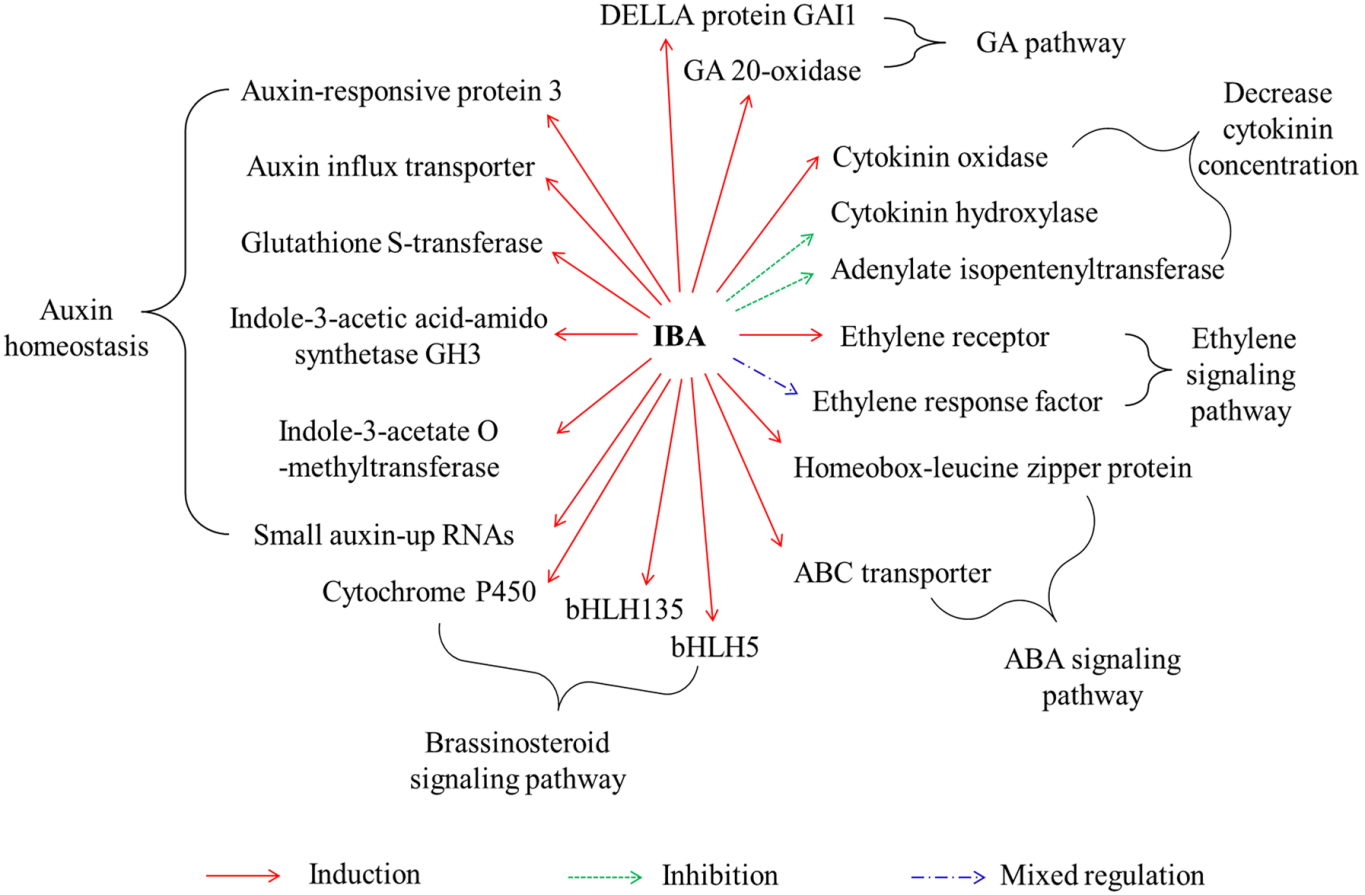
Summary of major differentially expressed genes involved in plant hormone signal transduction in tea cuttings.

Auxin interacts with many plant hormones to mediate developmental processes, such as cell division, elongation and differentiation [26]. For example, auxin and ethylene act synergistically to control root elongation, root hair and lateral root formation [27]. The role of gibberellins (GAs) in regulation of lateral root development is associated with GA signaling crosstalk with auxin [28]. Here, candidate genes involved in plant hormone signaling were identified and their roles in interactions among plant hormones were mapped (Figure 4).

Interestingly, genes associated with auxin homeostasis were the most seriously affected by IBA treatment. However, most induced genes were correlated with decreased free auxin. For example, indole-3-acetic acid-amido synthetases (GH3) can join free auxin to different amino acids and overexpression of GH3 in plants produces severe auxin-deficient phenotypes [29], [30]. Whereas indole-3-acetate O-methyltransferase modulates auxin homeostasis through methylation of auxin's free carboxyl group [31]. Here, expression of two GH3 and an indole-3-acetate O-methyltransferase were 157.0-, 91.7- and 29.7- fold induced by IBA, indicating mechanisms associated with auxin storage or degradation were initiated by exogenous IBA. Furthermore, these data suggest that not all auxin-induced genes act as positive regulators of AR formation, especially for those involved in auxin homeostasis.

The cytokinin, BR, abscisic acid (ABA), ethylene and GA pathways were also closely associated with IBA responses (Figure 4). Moubayidin and colleagues (2009) reported that organ differentiation largely depends on the cytokinin:auxin ratio [32]. High cytokinin content facilitates shoot formation and high auxin content promotes root formation. In this study, exogenous application of IBA greatly inhibited genes involved in cytokinin biosynthesis, such as adenylate isopentenyltransferase (comp89357_c0, 9.4 times lower than the control) and cytokinin hydroxylases (comp79128_c0 and comp47678_c0, 5.8 and 7.0 times lower than the control, respectively), but induced genes associated with cytokinin degradation, such as cytokinin oxidases (comp93557_c2 and comp55386_c0, 9.4 and 5.2 times higher than the control respectively). These results agree with previous findings and demonstrate that cytokinin content in basal parts of tea cuttings is decreased by IBA treatment. Also, of note, shoot development of tea cuttings was initially inhibited by IBA [10], which might be attributed to decreased cytokinin.

Auxin-BR interactions also are important to AR formation as these hormones modulate both cell expansion and proliferation and their transcriptional responses largely overlap [33]. Moreover, BR enhances classical auxin growth responses such as lateral root number [34] and gravitropic response [35]. In this study, five *bHLH* genes involved in BR signaling were up-regulated by IBA. Among them, four *bHLH135* genes were strongly homologous to Arabidopsis AT1G74500 (Table 4), which can stimulate BR signaling [25]. The high inducibility of *bHLH135* genes by IBA implicates essential roles in interactions between auxin and BR.

Furthermore, 7 genes involved in the ethylene pathway, 2 genes in the ABA pathway and 5 genes in the GA pathway were observed to be differentially expressed in this study, indicating a wide range of effects for IBA on plant hormone signal transduction.

### Secondary Metabolism

We previously identified that 2 genes involved in flavonoid biosynthesis were induced by IBA according to SSH data [11]. In this experiment, 55 genes associated with “secondary metabolites biosynthesis, transport and catabolism” were identified, broadening our understanding of the effects of IBA on the biosynthesis and distribution of secondary metabolites. Other than flavonoid biosynthesis, isoprenoid biosynthesis was also largely influenced by IBA.

Three genes (comp91052_c0 encoding 1-deoxy-D-xylulose-5-phosphate synthase 2, comp85717_c0 encoding 4-hydroxy-3-methylbut-2-enyl diphosphate reductase and comp48825_c0 encoding geranyl pyrophosphate synthase small subunit) involved in the 2-C-methyl-d-erythritol 4-phosphate (MEP) pathway were inhibited, while comp90840_c0 [3-hydroxy-3-methylglutaryl-coenzyme A reductase 1 (*HMG1*)] involved in the mevalonate (MVA) pathway was induced by IBA. The MVA pathway in cytoplasm and the MEP pathway in plastids are two distinct compartmentalized pathways involved in isoprenoid biosynthesis [36]. The MVA pathway is responsible for the synthesis of sterols, sesquiterpenes and the side chain of ubiquinone. In contrast, the MEP pathway provides the precursors for monoterpenes, sesquiterpenes, diterpenes, carotenoids and phytol group of chlorophylls [37]. Suzuki *et al.* (2004) reported that loss of function of *HMG1* in *Arabidopsis* causes dwarfing phenotype [38], which has 77% nucleotide sequence identity to the *HMG1* gene identified in this study. In contrast, plants treated with MEP pathway-specific inhibitors exhibit albino phenotypes, which are caused by preventing formation of β-carotene [39]. Further studies confirmed a function for the MVA pathway in plant development and a function for the MEP pathway in pigment formation [40]. Interestingly, our results indicate that IBA modulates the MVA and MEP pathways uniquely. The induction of MVA and the suppression of MEP by IBA facilitated cell growth but inhibited biosynthesis of carotenoids and chlorophylls in the basal part of tea cuttings, which might be important for AR formation and worthy of further study.

### Cell Wall Modification

Cell wall undergoes change during AR formation, specifically, in the basal parts of cuttings, it is degraded, loosened and stretched to facilitate cell elongation and division, which is needed for AR initiation [41]. In this study, 40 genes involved in cell wall organization were identified to be differentially expressed, which included 8 expansins and these were highly induced by IBA, data consistent with previous findings by Ludwig-Müller’s group (2005) [9]. Expansins are considered to be responsible for acid-induced cell wall loosening and are mainly expressed in rapidly growing tissues [9]. The potential function of these proteins are disrupting hydrogen bonds between cellulose microfibrils and promoting cell wall to loosen. As the wall rigidity declines, cells can elongate [42]. In addition to expansins, 7 cell wall associated genes are linked to auxin response and can modulate root development in the model plant *Arabidopsis*, namely pectinesterase (AT5G51500), galacturonosyl-transferase (AT3G58790), cellulase (AT1G71380), leucine-rich repeat extensin (AT1G62440), endoxyloglucan transferase (AT2G06850), cellulose synthase (AT4G18780) and invertase/pectin methylesterase inhibitor [43]. Here, 3 pectinesterase genes, a cellulase gene, 7 leucine-rich repeat extensin genes and 4 cellulose synthase genes were identified to be differentially expressed, indicating that cell wall modification is important to IBA-induced AR formation in tea cuttings. Moreover, many cell wall weakening genes were induced directly by IBA, suggesting that cell wall modification might not only be an early step of AR initiation, but also may be a limiting factor for determining timing of rooting in tea cuttings.

### Glutathione Metabolism

Glutathione homeostasis is crucial in auxin transport and root development [11], [44], [45]. The balance between the reduced (GSH) and oxidized forms (GSSG) of glutathione offers a thiol/disulfide buffer, which is affected by GSH oxidation, such as reactive oxygen species and by GSSG reduction with glutathione reductase. Here, we identified 8 differentially expressed genes involved in glutathione metabolism according to KEGG analysis (Table S3). Among them, a glutathione synthetase and two glutathione S-transferases (GSTs) were highly induced by IBA. Glutathione synthetase is the second enzyme in glutathione biosynthesis and GSTs may convert GSH to GSSG [46]. Our results illustrate that total glutathione and GSSG in tea cuttings may increase with IBA treatment, promoting the interaction between endogenous auxin and GSSG. In fact, exogenous application of GSSG did not affect the number of roots under normal conditions, but strongly enhanced rooting stimulatory effects of auxin treatment [47]. For example, a loss-of-function mutant of *AtGSTU17*, with a reduced GSH/GSSG ratio had fewer lateral roots in the presence of auxin [46]. Our results suggest that the interaction between auxin and GSSG might be necessary for AR formation of tea cuttings.

Our RNA-seq results also suggest other genes potentially involved in AR formation, such as those encoding calmodulin binding proteins [48], lipoxygenase [49] and proline-rich proteins [50]. These newly identified genes suggest a more global view for genes potentially participating in auxin-induced AR formation in tea cuttings. Although molecular functions of many genes remain unknown, our work offers a foundation for future characterization of gene functions to ascertain the metabolism of auxin-induced AR formation. Moreover, the numerous transcripts obtained here will facilitate genomic studies on *C. sinensis*.

## Supporting Information

Figure S1 **Species distribution of the NR annotated all-unigenes.**
(TIF)

Figure S2 **Gene Ontology classification of** ***C. sinensis*** **transcriptome.** Gene Ontology (GO) terms are summarized in three main categories: cellular component, molecular function and biological process. The left and right y-axes are indicating the percentage and the number of genes within a specific GO category, respectively.
(TIF)

Figure S3 **COG function classification of** ***C. sinensis*** **transcriptome.**
(TIF)

Figure S4 **KEGG pathway distribution of** ***C. sinensis*** **transcriptome.**
(TIF)

Table S1 **Primers used for quantitative real time RT-PCR.**
(DOCX)

Table S2 **The differentially expressed genes of tea cuttings treated with or without IBA.**
(XLS)

Table S3 **KEGG pathway analysis of differentially expressed genes of** ***C. sinensis*** **transcriptome.**
(XLSX)
