# Supplementary figures and images for: Transcriptome Analysis of Indole-3-Butyric Acid-Induced Adventitious Root Formation in Nodal Cuttings of Camellia sinensis (L.)
Source: PLoS One. 2014 Sep 12;9(9):e107201. doi: 10.1371/journal.pone.0107201 (PMC4162609; doi:10.1371/journal.pone.0107201)

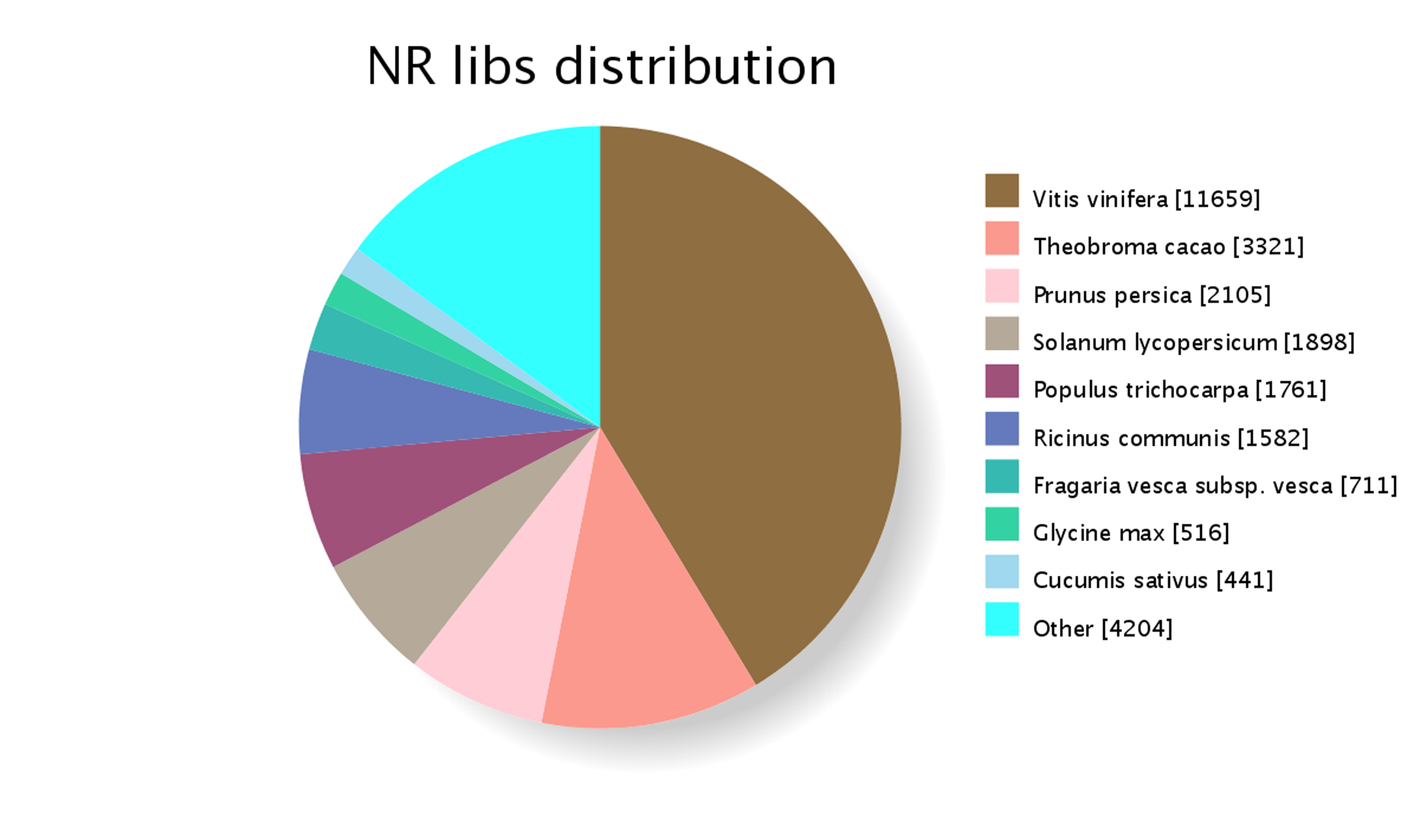

Supplement: Figure S1 — Species distribution of the NR annotated all-unigenes. (TIF) [file pone.0107201.s001.tif]

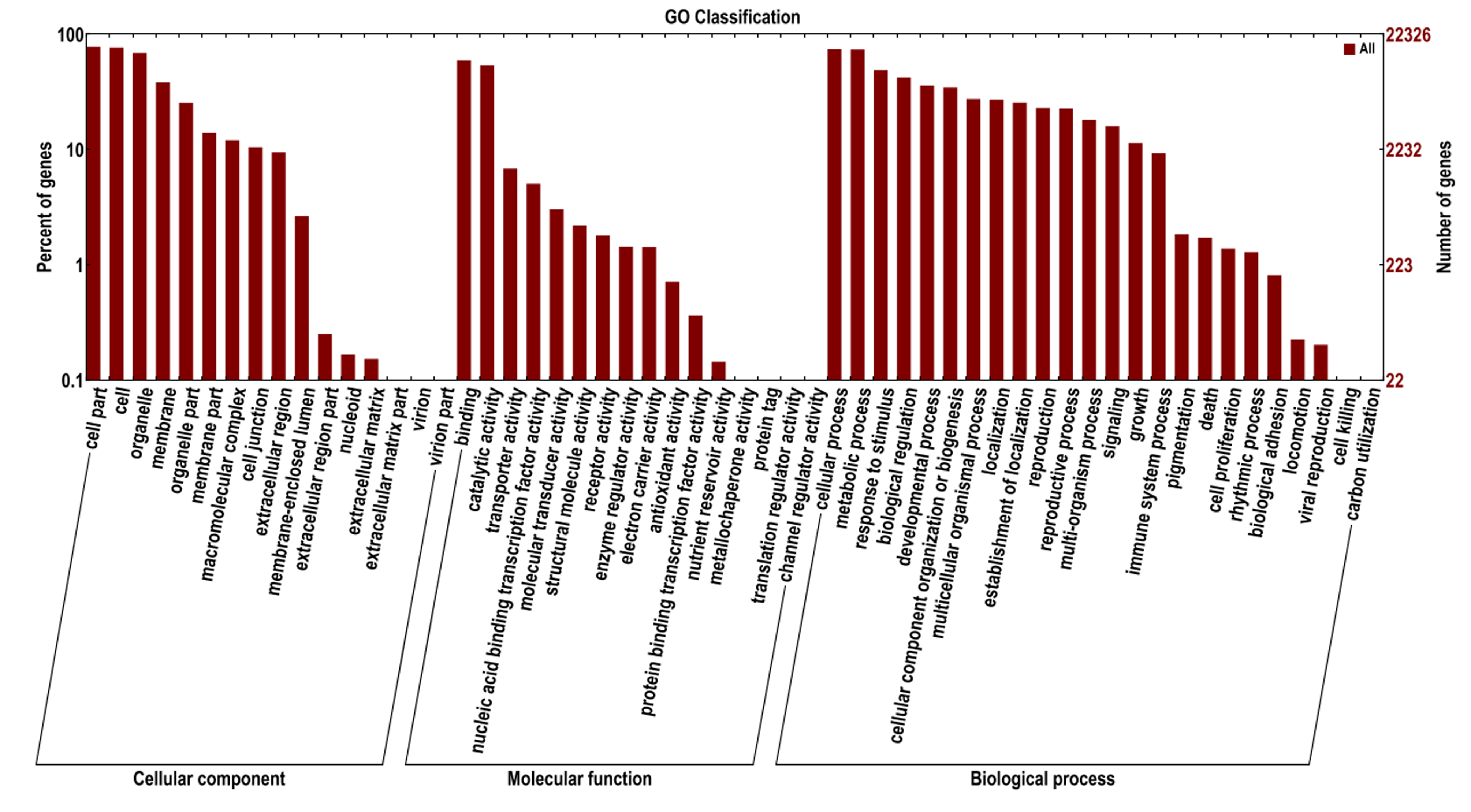

Supplement: Figure S2 — Gene Ontology classification of C. sinensis transcriptome. Gene Ontology (GO) terms are summarized in three main categories: cellular component, molecular function and biological process. The left and right y-axes are indicating the percentage and the number of genes within a specific GO category, respectively. (TIF) [file pone.0107201.s002.tif]

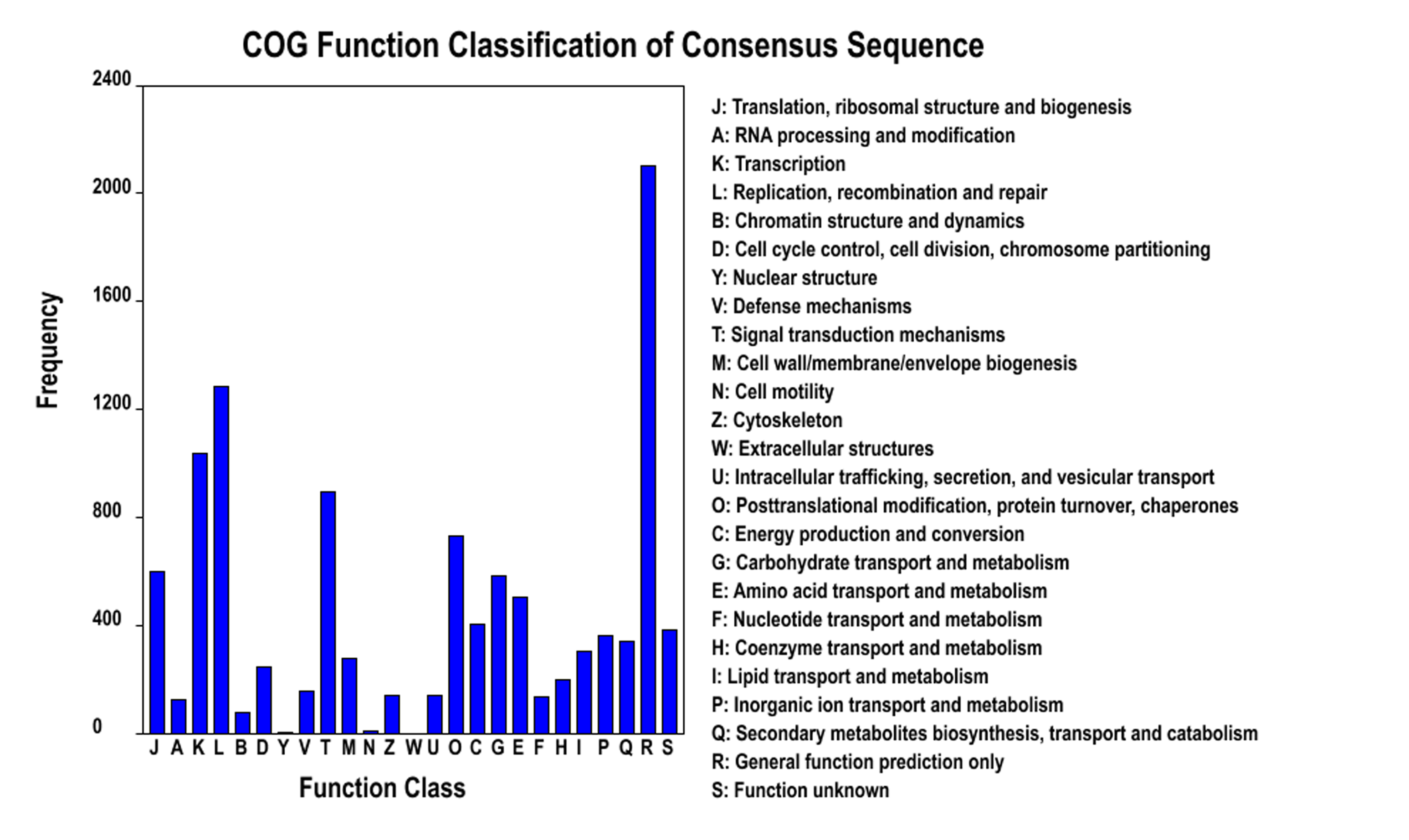

Supplement: Figure S3 — COG function classification of C. sinensis transcriptome. (TIF) [file pone.0107201.s003.tif]

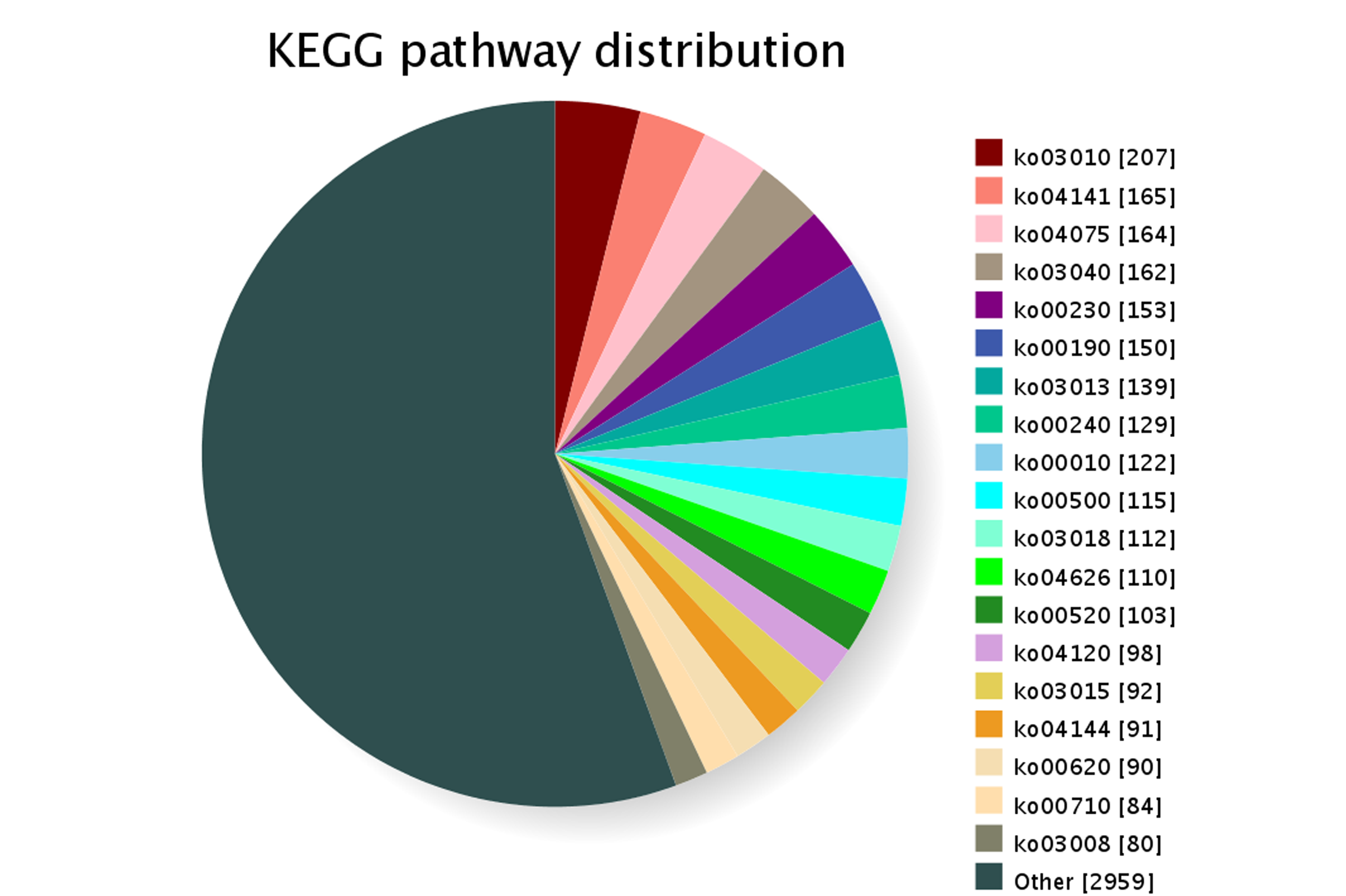

Supplement: Figure S4 — KEGG pathway distribution of C. sinensis transcriptome. (TIF) [file pone.0107201.s004.tif]
