# Supplementary material for: Transcriptome Analysis of Indole-3-Butyric Acid-Induced Adventitious Root Formation in Nodal Cuttings of Camellia sinensis (L.)
Source: PLoS One. 2014 Sep 12;9(9):e107201. doi: 10.1371/journal.pone.0107201 (PMC4162609; doi:10.1371/journal.pone.0107201)
Supplement: Table S1 — Primers used for quantitative real time RT-PCR. (DOCX) [file pone.0107201.s005.docx]

**Table S1. Primers used for quantitative real time RT-PCR**

| Gene no. | Functional annotation | Primer sequence(5’-3’) |
| --- | --- | --- |
| comp93023_c0 | NAD(P)-binding Rossmann-fold superfamily protein | F: AGTTGTGTTTATGAAGTGAACTATGCAT |
|  |  | R: CATGAGCTTCGAACTTCTTCCATATTAG |
| comp92737_c1 | Histidine kinase 2 | F: CAACAAGATTAGTAATTATCTGCCGGAA |
|  |  | R: CTTTTGATTTGCGTGATGTTCTTGATAA |
| comp94314_c0 | Glutamate synthase | F: CATATTTTAATGGAATGGCAGCAGAAAG |
|  |  | R: GTTGCTGTATCATCATTCTTAGTGTCAT |
| comp52177_c0 | 14 kDa proline-rich protein DC2.15 isoform 1 | F: TCACGAAATCAAATTACACACAAATCAC |
|  |  | R: AGCTTGCTTCTTAATGTTTGTGAGAAAA |
| comp52516_c0 | Beta-galactosidase 3 | F: TACATGTAATAGTTTTGTAAGCTACCGC |
|  |  | R: ATGGCTGTTTCTCAAAATATAGGTGTAC |
| comp82597_c1 | Auxin-responsive GH3 family protein | F: CATTTGGGTGTACATACTTTGGAATGAG |
|  |  | R: TCGTCAGTTGCTATACAGTCTTCTTATG |
| comp90888_c2 | Glutathione S-transferase L3 | F: AACTAACTTAGGGTCCTATTTGTCTACC |
|  |  | R: GAGTCTTGTTTTCCCTTGTACAATGTAT |
| comp93865_c0 | Isoflavone hydroxylase | F: GATCGTGATTAGTCCTTTGATGATTTCA |
|  |  | R: TTATCAGAGTTTTGAGAAGAATTTGGGG |
| comp92586_c0 | UDP-glycosyltransferase 73B4 | F: CCAACCTCTTATGATCAATCCTCTATCT |
|  |  | R: ATCTATATATGTTTCGGAAGTGTAGCCA |
| comp94649_c0 | Tonoplast dicarboxylate transporter | F: CATTTAAGGAGAGAGCTTGAAATCCTAG |
|  |  | R: TGTTGCTTCTTATTCGGAATTATGAACA |
| comp85853_c0 | DELLA protein GAI1-like isoform 1 | F: GATTCTTCGACTATTACAGGTACAGGTA |
|  |  | R: GTTTATTATTTCCATTTTCCCTCCTCGA |
| comp92938_c0 | Two-component response regulator ARR12 | F: CAGCGACTTTAGAATCAATATGTCTTCA |
|  |  | R: TGATTTCTTGCCATTATTCATTCTTTGC |
| comp89952_c0 | Beta-xylosidase | F: CAATTCTGATGTTACTGTCACTATGCTC |
|  |  | R: TTGAATTCTGTTTCTATCGATTGGTCAA |
| comp56873_c0 | Cytokinin dehydrogenase 7 | F: GAAATTATCCAATGCTGCATCAACAAC |
|  |  | R: CAATGGAGAGAAGCAGAAAATATTGGTA |
| comp87241_c0 | Cinnamate 4-hydroxylase | F: TAAAGTTGTTTCTATTGCAGCAACATTG |
|  |  | R: GTTGAAAGAAGAAGGAAAATAATGGCTG |
| comp94147_c0 | Lipoxygenase | F: AAACACTATTACCAAGATGCAAGCTTTA |
|  |  | R: GTTACCCAGATCATAGTTGTGAGAATTC |
| comp93352_c0 | Cyanidin-3-O-glucoside 2-O-glucuronosyltransferase | F: TGGTTAGTTTTGGCAGTCATATGTTTAA |
|  |  | R: CGATGTATTTTCCTTCAAGCTCTCTAAA |
| comp84014_c0 | Transcription factor bHLH135 | F: TCTAAGAATGTCAGAATCAACACCG |
|  |  | R: TGCCCAAGAAGAAGTATTAGTGAAG |
| comp94560_c0 | Transcription factor bHLH135 | F: CAAACTCCTAATTATGGATGCCTCA |
|  |  | R: AATAGGCGTTCTGACAAAGTATCTG |
| comp56004_c0 | Transcription factor bHLH135 | F: ATTGATGCTAATAGTCCTGAAGCTG |
|  |  | R: CCTTATCACCCCATATCATACATGT |
| comp126119_c0 | Transcription factor bHLH135 | F: TACAAAAGAGAGGATGATGAAGCTG |
|  |  | R: GGAGACTTGCAACTATATTAGGAAC |
| comp79682_c0 | Ornithine decarboxylase | F: GAGGACTTATTGTTCGACTGTGTTT |
|  |  | R: TAATCGCTGATGTATTGAACCCATT |
| comp88835_c0 | Sucrose transporter 3 | F: TACTCCTTCAACGCACTCAATAATT |
|  |  | R: AATGTTTACTGTGCGAATCTCAAGA |
| comp78422_c0 | Beta-1,3-galactosyltransferase 15 | F: ATTTCGTTCCATAGTTCCTTGTTCA |
|  |  | R: AGAACATTATTGACTTGGAAGCACT |
| comp87791_c3 | Callose synthase 3-like | F: TCCTCCTCTCTAAAACTGCTGATTA |
|  |  | R: GAGAGATTCGAACTTTAGGAATGCT |
| comp93557_c2 | Cytokinin oxidase | F: TTCATGCTCTTGTTCTTAAGCTGTA |
|  |  | R: GAGACAGACTTTGGATGTAGAACTG |
| comp55386_c0 | Cytokinin oxidase | F: AAACTCAGAACTATTCCATGCTGTT |
|  |  | R: CAATGACAAAGCCCTCAACATAATC |
| comp57485_c0 | Ethylene-responsive transcription factor 4 | F: CCATCTCAACATGTACAGATTCGAT |
|  |  | R: TTGTTTAAAAGGAAAATGGAACGCT |
| comp93549_c1 | Auxin-responsive protein IAA8 | F: AGATCACATAAGTTGCAGAAGTACC |
|  |  | R: GATGTTGCTATCCATCCACCAATAT |
| comp51706_c0 | Auxin-responsive protein IAA13 | F: ATCCAGTCTCCCTCTTTATCTTCAT |
|  |  | R: TTGGAAGAAAAGTGGATTTGAATGC |
| comp57530_c0 | F-box protein At2g27310-like | F: CACTGTTTAATTTGTCACTCATCCG |
|  |  | R: TGAGATGTGCTTGAAGATGAAGAAA |
| comp101272_c0 | Jasmonate-zim-domain protein 10 | F: ATTTTGAAAATTGCAAAGGGAGGAG |
|  |  | R: TTGATGTATTCTTCCCCGAAAATGT |
| comp83592_c0 | E3 ubiquitin-protein ligase PUB24 | F: ATCTTCAAACACATCCGAAATCTCA |
|  |  | R: CAGAAGGTGTTTATTTTCCGTTGTC |
| comp91945_c0 | ABC transporter C family member 10 | F: TCTTCCAACAATACCAATTTTGTGC |
|  |  | R: TATATGCACATACCAAGTGAAGCTC |
| comp89357_c0 | Adenylate isopentenyltransferase 3 | F: CGAGTATTACCCTTGATCTCCCTTA |
|  |  | R: TTGATTTGATGGTTGAGATGGGAAT |
| comp90244_c0 | Glutathione peroxidase 2 | F: GTTCTGATTGTGAATGTTGCTTCAA |
|  |  | R: GCGGTTTCCTGAATTTTCTCATTAG |
